# The Potential Prognostic Value of a Novel Hematologic Marker Fibrinogen-to-Lymphocyte Ratio in Head and Neck Adenoid-Cystic Carcinoma

**DOI:** 10.3390/jpm11111228

**Published:** 2021-11-19

**Authors:** Faris F. Brkic, Stefan Stoiber, Marlene Friedl, Tobias Maier, Gregor Heiduschka, Lorenz Kadletz-Wanke

**Affiliations:** 1 Department of Otorhinolaryngology and Head and Neck Surgery, Medical University of Vienna, 1090 Vienna, Austria; faris.brkic@meduniwien.ac.at (F.F.B.); marlene.friedl@hotmail.com (M.F.); tobias.maier@meduniwien.ac.at (T.M.); gregor.heiduschka@meduniwien.ac.at (G.H.); 2 Department of Pathology, Medical University of Vienna, 1090 Vienna, Austria; stefan.stoiber@meduniwien.ac.at; 3 Christian Doppler Laboratory for Applied Metabolomics, Medical University of Vienna, 1090 Vienna, Austria

**Keywords:** adenoid-cystic carcinoma, fibrinogen-to-lymphocyte ratio, prognostic marker, survival, outcome

## Abstract

Many patients with adenoid-cystic carcinoma (ACC) experience an indolent course of disease over many years but face late recurrence, and long-term survivors are rare. Due to its infrequent occurrence, it is hard to predict outcome in these patients. The fibrinogen-to-lymphocyte ratio (FLR) was recently proposed as an outcome prognosticator in different cancer entities. We aimed to investigate its prognostic relevance in patients with head and neck ACC. This retrospective analysis was performed including all patients treated for ACC between 1998 and 2020. The FLR ratio was calculated based on pretreatment values (0–7 days). The study cohort was dichotomized based on optimized threshold value and compared for differences in outcome (overall survival (OS) and disease-free survival (DFS)). In the cohort of 39 included patients, the OS was significantly longer in the low (*n* = 28) compared to the high pretreatment FLR group (*n* = 11) (median OS 150.5 months, 95% confidence intervals (CI) 85.3–215.7 months vs. 29.4 months, 95% CI not reached; *p* = 0.0093). Similarly, the DFS was significantly longer in the low FLR group (median DFS 74.5 months, 95% CI 30.6–118.4 months vs. 11.0 months, 95% CI 5.1–16.9 months; *p* = 0.019). The FLR is an easily obtainable and simple marker and may be a valuable outcome prognosticator in patients with ACC. Further studies are needed for validation of our results.

## 1. Introduction

Head and neck adenoid-cystic carcinoma (ACC) can be found in the major salivary glands, minor salivary glands of the tongue, cheek mucosa, lip, nasal cavity, and paranasal sinuses [1]. Overall, it is a rare tumor and accounts for about 1% of all head and neck malignancies [2]. However, within the group of salivary gland tumors, the ACC is fairly common. In particular, about 10% of major salivary gland malignancies are ACC [3]. Furthermore, it is the most common malignant tumor of the minor salivary glands and accounts for almost 60% minor salivary gland malignancies [1].

Regarding the outcome, mortality rates are usually low in the first years after diagnosis [4,5], and the spread to cervical lymph nodes is rarely observed [6]. Nevertheless, local control is a common concern, and multiple recurrences are frequently observed [5]. Finally, distant failure is another major issue in ACC patients, and the distant metastases are most often observed in the lung, liver, and bones via hematogenous spread [6].

Due to ACC’s infrequent occurrence and varying clinical course, it is very hard to estimate the outcome and plan the therapy for each individual case. Based on the inflammatory link between tumors, their microenvironment, and the systemic response [7], different inflammatory markers were analyzed for their prognostic potential in various cancer entities including head and neck malignancies. Studies have shown the correlation of survival outcome in patients with head and neck carcinoma with several different inflammation-associated markers such as the neutrophil-to-lymphocyte ratio (NLR) [8,9], platelet-to-lymphocyte ratio (PLR) [10], and C-reactive protein (CRP) [11]. In our previous study, we assessed the prognostic significance of certain inflammatory biomarkers in ACC and observed an association of outcome with NLR values [12].

The prognostic value of serum fibrinogen was already assessed in different cancer entities, with high pretreatment values correlating with worse outcomes in colorectal cancer as well as in lung cancer [13,14]. As noted by Zhang et al. [14], fibrinogen might reflect the tumor-specific inflammation response and may contribute to the proliferation of tumor cells. Furthermore, the absolute lymphocyte count is contained in several prognostic markers already showing prognostic significance in cancer patients, including the NLR and PLR [8,9,10]. Moreover, high pretreatment lymphocyte count was shown to correlate with better survival in gastric cancer. Indeed, it was proposed that lymphocytes might have a potent antitumor immune activity and play a role in tumor-associated immune response [15].

Based on these findings, it can be hypothesized that a ratio between serum fibrinogen and lymphocyte count could predict worse survival outcome in tumor patients. Indeed, the novel prognosticator fibrinogen-to-lymphocyte ratio (FLR) was recently proposed, and studies in esophageal cancer, gastric cancer, and non-small lung cancer attested the FLR’s prognostic value [16,17,18]. In particular, higher FLR values were associated with worse outcome.

Up until now, the prognostic significance of the FLR was evaluated neither in salivary gland carcinoma patients nor in ACC patients. Therefore, the aim of this study was to assess the prognostic value of the FLR in patients with ACC in regard to survival outcome. Moreover, the prognostic capacity of individual values of fibrinogen and absolute lymphocyte count was investigated as well.

## 2. Patients and Methods

This retrospective study was performed at the Department of Otorhinolaryngology, Head and Neck Surgery, at the Medical University of Vienna. All patients with histologically verified ACC that were initially treated at our institution between 1 January 1998 and 1 June 2020 were screened for availability of pretreatment values of absolute lymphocyte count and serum fibrinogen concentration. These were obtained from the routinely performed pretherapeutic blood samples (0–7 days prior to initial therapy). Patients with available values and follow-up data were included. Patients with a secondary malignancy or prior treatment were excluded. A total of 39 patients could be included in the study. The FLR was calculated by dividing the fibrinogen value (mg/dL) by the absolute lymphocyte count (G/L).

## 3. Statistics

R software (version 3.5.1, R Foundation for Statistical Computing, Vienna, Austria) was utilized for statistical analysis and graphical presentation. After calculating the FLR, the optimal threshold value (OTV) was determined in regard to OS. Based on the OTV, the study cohort was divided into the low and high FLR group. The log-rank test was utilized in order to compare OS and DFS between two patient groups. The compared survival times were presented using median and 95% confidence intervals (CI). The Kaplan–Meier curves were used for graphical illustration. Analogous to FLR, the survival analyses were performed for individual values of fibrinogen and absolute lymphocyte count as well.

## 4. Results

### 4.1. Patients

The median age of the cohort was 60.9 years (range 29.3–83.3 years). Twenty patients (51.3%) presented with a locally advanced primary tumor (T3 or T4). The majority of all patients (*n* = 31, 79.5%) had no radiologic or pathologic signs of regional lymph node metastases. A total of five patients (12.8%) harbored distant metastases at the time of initial work-up. The minor salivary glands were predominately the site of origin. Indeed, in twenty-four patients (61.5%), the tumors were located in the minor salivary glands. Table 1 shows the patient details and the respective tumor characteristics, for the whole cohort, as well as stratified for low and high pretreatment FLR.

Thirty-one patients (79.5%) were treated with surgery as a first-line treatment. Out of those, nine patients (23.1%) were subsequently treated with conventional postoperative radiotherapy, and six patients (15.4%) received adjuvant proton therapy. A single patient (2.6%) was treated with postoperative chemoradiotherapy. Primary radiotherapy or proton therapy was the selected treatment modality in five (12.8%) and two patients (5.1%), respectively. Concomitant chemotherapy was applied additionally in two patients (5.1%). Lastly, one patient (2.6%) was treated with palliative chemotherapy after initial diagnosis.

The median fibrinogen value was 373 mg/dL (range 215–731 mg/dL), and the median absolute lymphocyte count was 1.7 G/L (range 0.7–3.4 G/L) for the whole cohort. After calculation of the FLR, the median value of our study cohort was 223.2 (range 113.8–664.5). Furthermore, the OTV for FLR in regard to OS was calculated as 272.9. Based on this value, the patients were stratified into a low FLR group (FLR ≤ 272.9, *n* = 28) and a high FLR group (FLR > 272.9, *n* = 11). The OTVs for fibrinogen and absolute lymphocyte count in regard to OS were calculated as 350 mg/dL and 1.1 G/L, respectively. According to the respected OTV values, groups with low pretreatment values of fibrinogen and absolute lymphocyte count (≤OTV) contained 16 and 8 patients, respectively.

### 4.2. Survival

Eleven out of 39 patients (28.2%) died, and locoregional or distant recurrence was observed in 20 patients (51.3%) during follow-up. A total of ten patients (25.6%) were diagnosed with more than one recurrence during their course of disease. Median OS and DFS measured 39.7 months (range 0.4–163.1 months) and 30.4 months (range 0.4–127.7 months), respectively.

**Figure 1:**
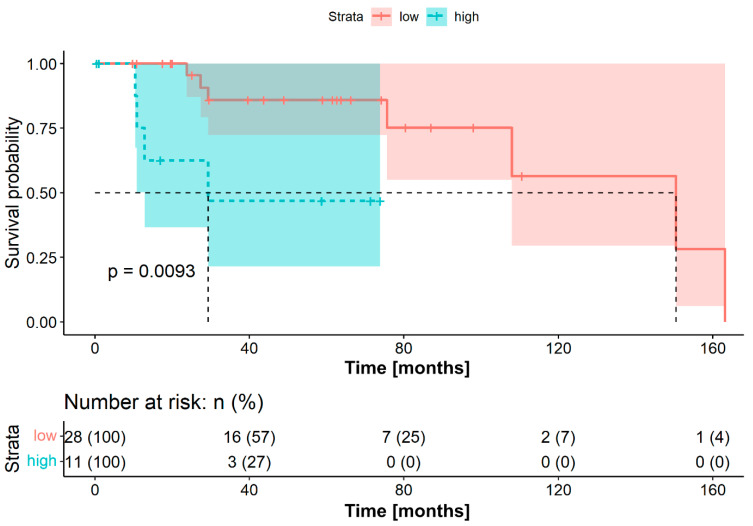
Overall survival for patients with adenoid-cystic carcinoma stratified into a low (*n* = 28) and high group (*n* = 11) according to the optimal threshold for fibrinogen-to-lymphocyte ratio for overall survival (272.9). The corresponding brighter colored areas represent the 95% confidence intervals. The survival times were statistically significantly different between patients with low and high fibrinogen-to-lymphocyte ratio (log-rank test *p* = 0.0093).

**Figure 2:**
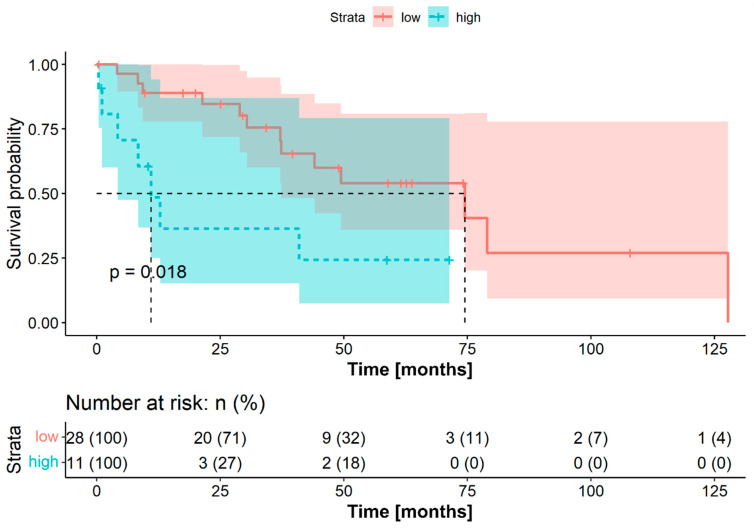
Disease-free survival for patients with adenoid-cystic carcinoma stratified into a low (*n* = 28) and a high group (*n* = 11) according to the optimal threshold for fibrinogen-to-lymphocyte ratio for overall survival (272.9.). The corresponding brighter colored areas represent the 95% confidence intervals. The survival times were statistically significantly different between patients with low and high fibrinogen-to-lymphocyte ratios (log-rank test *p* = 0.018).

As noted previously, the cohort was dichotomized into low and high FLR groups based on the FLR OTV. The OS was significantly longer in the group with low preoperative FLR (median OS 150.5 months, 95% CI 85.3–215.7 months vs. 29.4 months, 95% CI not reached; log-rank test *p* = 0.0093). Similarly, the DFS was significantly prolonged in the low FLR group (median DFS 74.5 months, 95% CI 30.6–118.4 months vs. 11.0 months, 95% CI 5.1–16.9 months; log-rank test *p* = 0.018). Figure 1 and Figure 2 show the Kaplan–Meier survival curves for patients with low and high FLR, for OS and DFS, respectively.

Furthermore, low serum fibrinogen was associated with prolonged survival times as well. However, these results were not statistically significant (median OS 150.5 months, 95% CI 42.3–258.7 months vs. 107.9 months, 95% CI not reached; *p* = 0.090 and median 74.5 months, 95% CI 18.7–130.3 months vs. 44.1 months, 95% CI 30.7 vs. 57.5 months; *p* = 0.14). On the other hand, high absolute lymphocyte count was significantly associated with longer OS and DFS (150.5 months, 95% CI 81.8–219.2 months vs. OS 29.4 months, 95% CI 0.0–65.7 months; *p* = 0.025 and median 74.5 months, 95% CI 30.0–119.0 vs. 8.4 months, 95% CI 0.4–16.4 months; *p* = 0.004).

## 5. Discussion

Based on three prior studies, showing the prognostic significance of FLR in esophageal [16], gastric [17], and non-small lung cancer [18], we wanted to examine the role of FLR in ACC. The current study revealed a potential link between pretherapeutic FLR and survival outcome in patients with ACC. In particular, high pretreatment FLR was statistically significantly associated with worse OS and DFS.

The role of cancer-associated systemic inflammation as well as inflammation of the tumor microenvironment is well investigated [19]. Furthermore, tumor-related hypercoagulative and prothrombotic states are other widely discussed phenomena [20]. Fibrinogen is known as an important coagulation factor as well as an acute-phase protein [21]. Therefore, fibrinogen is a marker that may reflect both inflammation and the hypercoagulative state in cancer patients. Moreover, fibrinogen facilitates tumor cells evading natural killer cells, as noted by Zhang and collaborators [14]. Several authors assessed the value of pretreatment fibrinogen value as an outcome prognosticator in patients with different cancer entities. In particular, the high fibrinogen was shown to be associated with poor survival in gastric cancer [22]. Similarly, its unfavorable prognostic value was reported in urothelial and colon cancer [13,23]. Furthermore, the role of fibrinogen in tumor metastasis is known, and this could be demonstrated in an in-vitro study assessing lung cancer [24]. Moreover, Tang et al. noted the association of high preoperative values of fibrinogen with the colorectal cancer metastases [25]. In our cohort, no significant association of the pretherapeutic serum fibrinogen with survival outcome was observed.

The second component of the FLR is the absolute lymphocyte count. Lymphocytes are important antineoplastic factors and play an important role in cancer-specific immune response [17]. In detail, they have direct, cytotoxic effects on tumor cells and are able to suppress tumor cell proliferation via humoral and cytokinetic pathways [19]. Isolated lymphocytosis was reported to be a positive prognosticator in tumor patients by several authors. In particular, Miyoshi et al. [26]. reported that high absolute lymphocyte count associated with improved OS in patients with metastatic breast cancer treated with eribulin. Another group noted that low absolute lymphocyte count combined with high absolute monocyte count independently predicted worse OS and DFS in gastric cancer [27]. Similarly, low preoperative lymphocyte count was associated with worse survival in patients with oral cancer [28]. In the present study, we were able to present significant association of the high pretreatment absolute lymphocyte count with longer OS and DFS.

The FLR represents a combination of these two markers and should synergistically enhance their individual prognostic value. Furthermore, it potentially reflects the cross-talk between inflammation and coagulation in cancer [16]. Herein, we reported on the prognostic significance of the FLR on the survival in patients with ACC, and our findings were similar to other reports on different cancer types. Fan et al. [16]. showed worse OS and DFS in patients with esophageal squamous cell carcinoma after radical esophagectomy with elevated preoperative FLR. Similarly, poorer OS was observed in non-small cell lung cancer patients with high FLR in a study of Liu et al. [18]. The negative prognostic value of FLR was even shown in patients with gastric cancer. In particular, high FLR values were associated with increased rates of peritoneal dissemination [17].

Up to date, only a handful of studies assessed the prognostic significance of different hematologic and inflammatory markers in ACC. In a previous study, we have shown that high NLR predicts the occurrence of multiple reoccurrences [12]. Furthermore, Damar et al. [29] analyzed pretreatment values of the neutrophil-to-lymphocyte ratio in patients with salivary gland tumors including ACC. They noted that the NLR could potentially be used in order to distinguish between low- and high-grade salivary gland tumors. Therefore, our current data might add some useful information to our understanding of the relevance of serum biomarkers in ACC.

There are some limitations concerning our study. First, the number of included patients with available follow-up data and pretreatment values of serum fibrinogen and absolute lymphocyte count was limited. Based on this, we were not able to perform a subgroup analysis and evaluate for potential confounders (e.g., different therapy approaches or age). However, as the ACC is a rare entity^1^, the number of patients included in our study is comparable or even higher than many of the clinical studies assessing head and neck ACC [30,31,32]. Furthermore, since the data is scarce and heterologous in regard to optimal cut-off values for FLR, we determined it by calculating the OTV for OS, which was used for patient stratification. Validation of this cut-off value in an external cohort is warranted. Another important limitation is the retrospective study design.

## 6. Conclusions

Our study provides first insights into the potential prognostic value of the FLR in patients with head and neck ACC. Since its calculation is based on values of routinely performed blood tests prior to cancer therapy, it represents a simple and easily obtainable outcome prognosticator. However, further studies with larger patient cohorts are mandatory for validation of our results.

## Figures and Tables

**Table 1:** Patient demographics, tumor characteristics with treatment approaches. n; number of patients, FLR; fibrinogen-to-lymphocyte ratio, PORT; postoperative radiotherapy or photon therapy, PORCHT; postoperative chemoradiotherapy or postoperative chemotherapy and photon therapy, RCHT; primary chemoradiotherapy or primary photon therapy with concomitant chemotherapy.

	All Patients		Low FLR Group		High FLR Group	
*n*, %	39	100	28	71.8	11	28.2
Age, year						
Median	60.9		61.1		60.6	
(Range)	29.3–83.3		29.3–83.3		31.4–79.8	
Age, by decade	n	%	n	%	n	%
20–30	1	2.6	1	3.6	0	0
30–40	6	15.4	4	14.3	2	18.2
40–50	5	12.8	2	7.1	3	27.3
50–60	6	15.4	6	21.4	0	0
60–70	13	33.3	10	35.7	3	27.3
70–80	7	17.9	4	14.3	3	27.3
80–90	1	2.6	1	3.6	0	0
Gender	n	%	n	%	n	%
Male	20	51.3	14	50	6	54.5
Female	19	48.7	14	50	5	45.5
T classification	n	%	n	%	n	%
T1	4	10.3	3	10.7	1	9.1
T2	15	38.5	13	46.4	2	18.2
T3	7	17.9	5	17.9	2	18.2
T4	13	33.3	7	25	6	54.5
N classification	n	%	n	%	n	%
N0	31	79.5	23	82.1	8	72.7
N1	1	2.6	0	0	1	9.1
N2	5	12.8	3	10.7	2	18.2
N3	2	5.1	2	7.1	0	0
M classification	n	%	n	%	n	%
M0	34	87.2	26	92.9	8	72.7
M1	5	12.8	2	7.1	3	27.3
Primary therapy	n	%	n	%	n	%
Surgery	31	79.5	23	82.1	8	72.7
PORT	15	38.5	10	35.7	5	45.5
PORCHT	1	2.6	0	0	1	9.1
Radiotherapy	7	17.9	5	17.9	2	18.2
RCHT	2	5.1	2	7.1	0	0
Palliative chemotherapy	1	2.6	0	0	1	9.1
Localisation	n	%	n	%	n	%
Parotid gland	7	17.9	4	14.3	3	27.3
Submandibular gland	7	17.9	5	17.9	2	18.2
Sublingual gland	1	2.6	1	3.6	0	0
Minor salivary glands	24	61.5	18	64.3	6	54.5

## Data Availability

The data that support the findings of this study are available on request from the corresponding author, [L.K.-W.].
